# Transgender health content in medical education: a theory-guided systematic review of current training practices and implementation barriers & facilitators

**DOI:** 10.1007/s10459-022-10112-y

**Published:** 2022-04-12

**Authors:** Jason van Heesewijk, Alex Kent, Tim C. van de Grift, Alex Harleman, Maaike Muntinga

**Affiliations:** 1 Center of Expertise on Gender Dysphoria, Amsterdam University Medical Center, Location VUmc, De Boelelaan 1131, 1081 HX Amsterdam, The Netherlands; 2 Faculty of Health Sciences, Simon Fraser University, Burnaby, Canada; 3 Departments of Plastic, Reconstructive and Hand Surgery and of Psychosomatic Gynecology and Sexology, Amsterdam University Medical Center, Location VUmc and AMC, Amsterdam, The Netherlands; 4 Centre for Sexual Health, GGD Amsterdam, Amsterdam, The Netherlands; 5 Department of Ethics, Law and Humanities, Amsterdam University Medical Center, Location VUmc, Amsterdam, The Netherlands

**Keywords:** Gender identity, Health equity, LGBT+, Medical education, Queer theory, Systematic review, Transgender health, Trans-inclusive healthcare, Training interventions, Transgender pedagogy

## Abstract

Health disparities faced by transgender people are partly explained by barriers to trans-inclusive healthcare, which in turn are linked to a lack of transgender health education in medical school curricula. We carried out a theory-driven systematic review with the aim to (1) provide an overview of key characteristics of training initiatives and pedagogical features, and (2) analyze barriers and facilitators to implementing this training in medical education. We used queer theory to contextualize our findings. We searched the PubMed/Ovid MEDLINE database (October 2009 to December 2021) for original studies that reported on transgender content within medical schools and residency programs (*N* = 46). We performed a thematic analysis to identify training characteristics, pedagogical features, barriers and facilitators. Most training consisted of single-session interventions, with varying modes of delivery. Most interventions were facilitated by instructors with a range of professional experience and half covered general LGBT+-content. Thematic analysis highlighted barriers including lack of educational materials, lack of faculty expertise, time/costs constraints, and challenges in recruiting and compensating transgender guest speakers. Facilitators included scaffolding learning throughout the curriculum, drawing on expertise of transgender people and engaging learners in skills-based training. Sustainable implementation of transgender-health objectives in medical education faces persistent institutional barriers. These barriers are rooted in normative biases inherent to biomedical knowledge production, and an understanding of categories of sex and gender as uncomplicated. Medical schools should facilitate trans-inclusive educational strategies to combat transgender-health inequities, which should include a critical stance toward binary conceptualizations of sex and gender throughout the curriculum.

**Supplementary Information:**

The online version contains supplementary material available at 10.1007/s10459-022-10112-y.

## Introduction

Transgender individuals are increasingly asserting visibility in all areas of social life. Transgender is an umbrella term to refer to individuals whose birth-assigned sex and gender do not align. ‘Sex’ is generally used to describe biological characteristics of being male, female or intersex, often referred to as the ‘3G’ axes (genes, gonads, genitals) (Fausto-Sterling, 2012), whereas ‘gender’ indicates social aspects of being assigned male, female or intersex at birth—with the recognition that some cultures acknowledge a wider spectrum of gender identities (Carrier et al., 2020). Gender generally refers to expressions, roles and relationships associated with maleness and femaleness, as well as to gender identity, the ‘felt’ experience of one’s own gender (Gilbert, 2009; Martin, 2004; Oliffe and Greaves, 2011). While sex as a construct is generally considered to consist of stable and fixed categories, gender is generally understood as culturally-constructed, and experiences of gender are spectral, unstable and fluid (American Psychiatric Association, 2013). Transgender people might use a range of identity labels to describe and express their gender, such as (transgender) male or (transgender) female, nonbinary, gender queer, gender fluid, agender, two spirit or other culturally- and linguistically- situated terms (Pruden, 2021), and so on. Some, but not all, transgender people experience gender dysphoria (Sudenkaarne, 2020). Gender dysphoria has been defined as significant distress or problems in functioning someone experiences due to an incongruence between the experienced/expressed gender and the assigned sex at birth (American Psychiatric Association, 2013).

The medical and health care field is uniquely equipped to support transgender individuals in their mental and physical wellbeing. Although medically transitioning is not desired by all and not (fully) available to all, an increasing number of people who identify under the transgender umbrella choose to enter a medically-assisted transition trajectory in addition to their social transition (Wiepjes et al., 2018). Insight in the health impact of gendered minority stress has increased understanding in the pre- and post- transition psychological needs of transgender individuals (Hendricks & Testa, 2012). At the same time, it has been well-documented that transgender communities face unique health risks and persistently experience poorer health outcomes when compared to their cisgender counterparts (i.e., people whose gender identity aligns with the gender assigned to them at birth). Reported health inequities include increased rates of cancer, poorer cardiovascular health, and more chronic diseases, sexually-transmitted infections, substance misuse and mental health concerns (Lo & Horton, 2016; Park & Safer, 2018; Streed et al., 2019; Taylor et al., 2018; Underman et al., 2016).

Several explanations for these disparities have been proposed. One is the fact that transgender individuals are confronted with persisting societal inequities and injustices, including in healthcare (Lo & Horton, 2016). A common concern among advocates for transgender health is that transgender people globally face barriers to accessing appropriate, safe and quality medical care. Many of these barriers are located at the level of the health system itself. Obstacles include physician unwillingness to treat transgender patients (Eriksson & Safer, 2016), physicians’ lack of knowledge on and experience with transgender medicine (Braun et al., 2017; Click et al., 2019; Eriksson & Safer, 2016; Greene et al., 2017; Kidd et al., 2016; Safer & Pearce, 2013; Streed et al., 2019; Vance et al., 2017, 2018), perceived or actual discrimination and/or transphobia (i.e., bigotry and prejudice targeting transgender people) in healthcare encounters (Braun et al., 2017; Cherabie et al., 2018; Greene et al., 2017; Kidd et al., 2016; Salkind et al., 2019; Sawning et al., 2017; Taylor et al., 2018; Underman et al., 2016), as well as cisgender bias embedded in healthcare settings and systems (Kidd et al., 2016; Taylor et al., 2018). Specific examples of harmful encounters with healthcare professionals include transgender patients reporting being addressed by the wrong names and pronouns, being asked inappropriate and unrelated questions, having the severity of their concerns underestimated or overshadowed, being confronted with hurtful or insulting language, having their gender identity belittled or ridiculed, being denied care, and feeling that they have to educate healthcare professionals (Braun et al., 2017; Salkind et al., 2019; Underman et al., 2016). These barriers lead to transgender individuals avoiding health care, delaying care until urgent or emergency situations arise or seeking hormones and/or surgeries from unregulated and unmonitored sources (Braun et al., 2017; Cherabie et al., 2018; Sawning et al., 2017; Underman et al., 2016). The lack of appropriate care and discrimination in healthcare settings combines with the compounding effects of minority stress from stigma and discrimination in society (Cherabie et al., 2018; Kidd et al., 2016; Streed et al., 2019).

### Transgender health and medical education

Part of the problem enabling physicians’ lack of knowledge and reinforcing discriminatory attitudes in patient-provider interactions is the inadequate education and training for health professionals in transgender medicine (Click et al., 2019; Eriksson & Safer, 2016; Fung et al., 2020; Kidd et al., 2016; Leandre et al., 2021; Marshall et al., 2017; Muntinga et al., 2020; Safer & Pearce, 2013; Underman et al., 2016; Vance et al., 2017, 2018). Data on training for health professionals in transgender medicine is only slowly becoming available, because studies tend to examine lesbian, gay, bisexual, transgender plus (LGBT+) health collectively in medical education, rather than focusing on transgender-related topics specifically (Braun et al., 2017; Cherabie et al., 2018; Click et al., 2019; Cooper et al., 2018; Kidd et al., 2016). Recent studies conclude that, while the number of context-tailored educational intervention formats are expanding, there is limited evidence to suggest that education interventions are translating into improved care or improved clinical outcomes for transgender patients, and that so far no best practice for curricular development has been identified (Nolan et al., 2020). Moreover, literature continuously suggests that physicians have misconceptions and anxiety around the legitimacy and safety of medical interventions for gender transition (Safer & Pearce, 2013; Vance et al., 2017, 2018), and further lack the knowledge, skills and exposure to provide gender-affirming care (Fung et al., 2020; Taylor et al., 2018). Finally, most of the current research reports from the US and Canada (Martins et al., 2020).

Hence*,* to ensure that transgender people benefit from current therapeutic options and to counter the medical system’s role in the reproduction of transgender health inequities, (future) physicians should harness adequate knowledge and skills, as well as critical gender consciousness. In the context of an increasing number of people living under the transgender umbrella and the previously stated adversities they face receiving healthcare, medical students should be provided adequate training in transgender health and gender and health, including exposure to frameworks that problematize the understanding of gendered bodies as stable and fixed as opposed to spectral and fluid (Barcelos, 2019; Pregnall et al., 2021). International organizations such as the World Health Organization (2013) and medical professional organizations such as the Institute of Medicine (2011), the Joint Commission and the US Department of Health and Human Services (2011), the American Medical Association (2016), the American Psychiatric Association (2012) and the Association of American Medical Colleges (AAMC) (Hollenbach et al., 2014) recommend enhanced training around LGBT+health, with dedicated attention to transgender health, in medical education. The Association of American Medical Colleges’ recommendations set forth in “Implementing Curricular and Institutional Climate Changes to Improve Health Care for Individuals Who Are LGBT, Gender Nonconforming (GNC), or Born with Differences in Sex Development (DSD)” has been profoundly influential as an implementation standard and practical resource for medical educators (Hollenbach et al., 2014). However, while these organizations have been advocating for transgender health content in medical education for nearly a decade, there is no requirement to do so or structures of accountability through accreditation bodies; therefore, the onus is on individual medical schools to take up these calls to action (Streed et al., 2019).

In light of the current circumstances and the pivotal moment in history in which we find ourselves, there is a need to synthesize available literature to capture the current state of training related to transgender health in medical student curricula and residency programs worldwide. Such synthesis aids in formulating medically-relevant, transgender health justice-oriented, and sustainable recommendations for evidence-based practices in transgender health education.

### Aim of the study

In this theory-guided systematic review, we aim to assess the current state of training related to transgender health in medical student curricula and residency programs worldwide. We provide an overview of key characteristics of current training initiatives in medical student curricula and residency programs, and analyze barriers and facilitators to implementing this training. By weaving theory throughout our systematic review, we aim to highlight which parts of transgender health education need further study and how current pedagogical approaches could be advanced. Ultimately, we aim to support the advancement of transgender-equitable health care by strengthening health systems’ capacity to uphold and integrate appropriate treatment and care for transgender people.

### Theoretical lens: queer theory

This review and the articles selected for inclusion are situated within discourses and explanatory models of gender that have implications for health professional training in transgender medicine. Queer theory is concerned with a critical and counter-hegemonic analysis of gender and sexuality. It particularly questions the normative reproduction of gender, sex and sexuality in science and society. Queer theory initially emerged as a way of thinking in the field of humanities and cultural studies, but eventually evolved towards its own interdisciplinary field of thought (Hall & Jagose, 2012; Jagose, 1996). The analytical viewpoints that unite queer theorists are an amalgamation of critical ideas that seek to expose, disrupt and transform dominant ideologies about gender, sex and sexuality at the interpersonal and structural levels. While a definition of queer would defeat its own purpose, in his famous work ‘Cruising Utopia’ José Esteban Muñoz connects queer imagination to “a failure to be normal” (Muñoz, 2019, p. 172). Indeed, then, the core objective of scholars using queer theory is to critically question ideas of normalcy, to foreground complexity, and to propose new ways of understanding sexuality and gender through centering bodies and experiences considered ‘deviant’ by the mainstream. The work of constructivist philosophers Michel Foucault and Judith Butler have particularly been instructive in shaping queer theoretical thought. Both argue that sexuality (Foucault, in his History of Sexuality) and gender (Butler, in ‘Gender Trouble’ and ‘Bodies that Matter’) are not fixed biological realities, but socially-constructed systems produced by everyday human interactions, and that normative notions of gender, sex and sexuality – such as the idea that heterosexuality is natural, or that sex and gender are binaries – are kept in place by power dynamics at the socio-structural level (Butler, 1990, 1996; Foucault, 1978). These ideas have been built on by scholars in the natural sciences, such as biologist Anne Fausto-Sterling and theoretical physicist Karen Barad (e.g., Barad, 2015; Fausto-Sterling, 2019).

In recent decades, queer critiques of biomedical systems of knowledge have been increasing, most notably coming from scholars in the field of cultural studies, gender studies, and disability studies (Hilary Malatino, 2019; Jones, 2020; McRuer, 2006; Spade, 2006). However, as a theoretical framework, queer theory has only been sparsely used within the medical and health sciences. Its tenets show up in the context of health inequities (e.g., Reid & Ritholtz, 2020) or as social science critiques of medical practices and knowledge, for instance in the field of medical and health humanities, medical anthropology, and bioethics (Moesch, 2019; Robertson, 2017; Spurlin, 2019; Wahlert & Fiester, 2012). These authors use queer theory to expose the hetero-centrist and cis-centrist biases (i.e., positioning heterosexuality and cisgender identity as the unquestioned norms), as well as binary norms that shape and underlie the production of biomedical knowledge about bodies, behaviors and orientations. They argue, for instance, that the pathologization of queer lives inherent to medical dualist epistemology results in suboptimal care for LGBT+—patients, both through unnecessary medicalization and insufficient care (Eckhert, 2016).

When ‘queer’ is used in post-graduate and graduate medical education research, it refers to curriculum gaps, patient identities in relation to culturally safe care and physician competencies, and the experiences of LGBT+-students (Baker & Beagan, 2014; Donald et al., 2017; MacCormick & George, 2020; Muntinga et al., 2020; Streed et al., 2019; Tollemache et al., 2021). Applying a queer lens to medical education has led authors to suggest the existence of a global discursive and systemic invisibility of queer narratives and needs, and a widespread refusal to label sexual and gender minority status as medically significant – a situation Müller (2018) identifies as an active process of erasure. As such and albeit in small volumes, queer theoretical work performed from inside the medical institutions has revealed how non-normative bodies and experiences are simultaneously marked as deviant and erased by the medical institution, including medical education (Feder, 2009; Iroegbulem, 2020; Meer & Müller, 2017; Müller, 2018; Robertson, 2017). Queer theory, therefore, is emerging as a useful analytic framework for understanding and questioning complex processes of standardization and exclusion in health care and education, and as such can be understood as a means of resistance against the social hierarchies that underpin these processes (Sifuentes, 2021).

Because queer approaches seek to transform current logics of medical knowledge systems, their use allows medical educators to critically question medical ontologies and epistemologies of gender, sex and sexuality, and to challenge the use of these constructs as uncomplicated clinical and research categories that inform everyday medical practice. In this theory-guided review about transgender health content in medical education, we apply a queer theoretical approach to extract deeper meaning when interpreting and contextualizing our findings. By understanding current efforts to implement transgender health content in medical education through a queer theoretical lens, we aim to identify trans-inclusive teaching strategies at all levels of the medical education system, as well as expose the institutional norms that function as barriers to implementation of these strategies, thus hindering transformation toward transgender-equitable health care.

## Methods

### Theory-guided literature review

We used a novel, theory-driven approach to focus the methods used in this review to respond to the knowledge question and to unpack the complex phenomenon that is gender diversity and health education. Although evidence synthesis efforts in health professions education are not often guided by theory, it has been suggested that the use of conceptual and theoretical frameworks facilitates the identification of shortcomings in the review’s included studies, literature gaps, and missing links in the body of literature (Buhi & Goodson, 2007; Godfrey et al., 2010). By viewing the results of this review through a queer theoretical analytical frame, we aim to emphasize areas of medical education, in particular areas where gender and sexuality are concerned, that might benefit from further study, development and advancement.

### Search strategy

This theory-guided systematic review was performed according to the Preferred Reporting Items for Systematic Reviews and Meta-Analyses (PRISMA) 2020 statement (Page et al., 2021). A systematic literature search using the PubMed database was conducted and updated for the date range October 2009 to December 2021 using the following keywords: for transgender individuals (“transgender persons”, “gender identity”, "gender nonconform*", "gender identity disorder", "gender dysphor*", "gender incongruen*", "gender varian*", "gender ambiguity", “transgender”, “transsexual*”, “lgbt*”, "sexual and gender minorities", "sexual minorit*”, "gender minorit*"); for education (“education, medical", "medical education", "health education", "medical curriculum", "schools, medical", "students, medical", "medical schools", “residen*”); and for training ("inservice training", "health training", “teaching”, "learning module", "diversity training", “e-learning”, "training needs"). The “OR” operator was used within and the “AND” operators between categories. Lastly, the filter ‘English’ was applied. This search resulted in 766 articles. Three additional articles were found through screening of reference sections and recommendations by experts in the field.

### Eligibility criteria

**Fig. 1:**
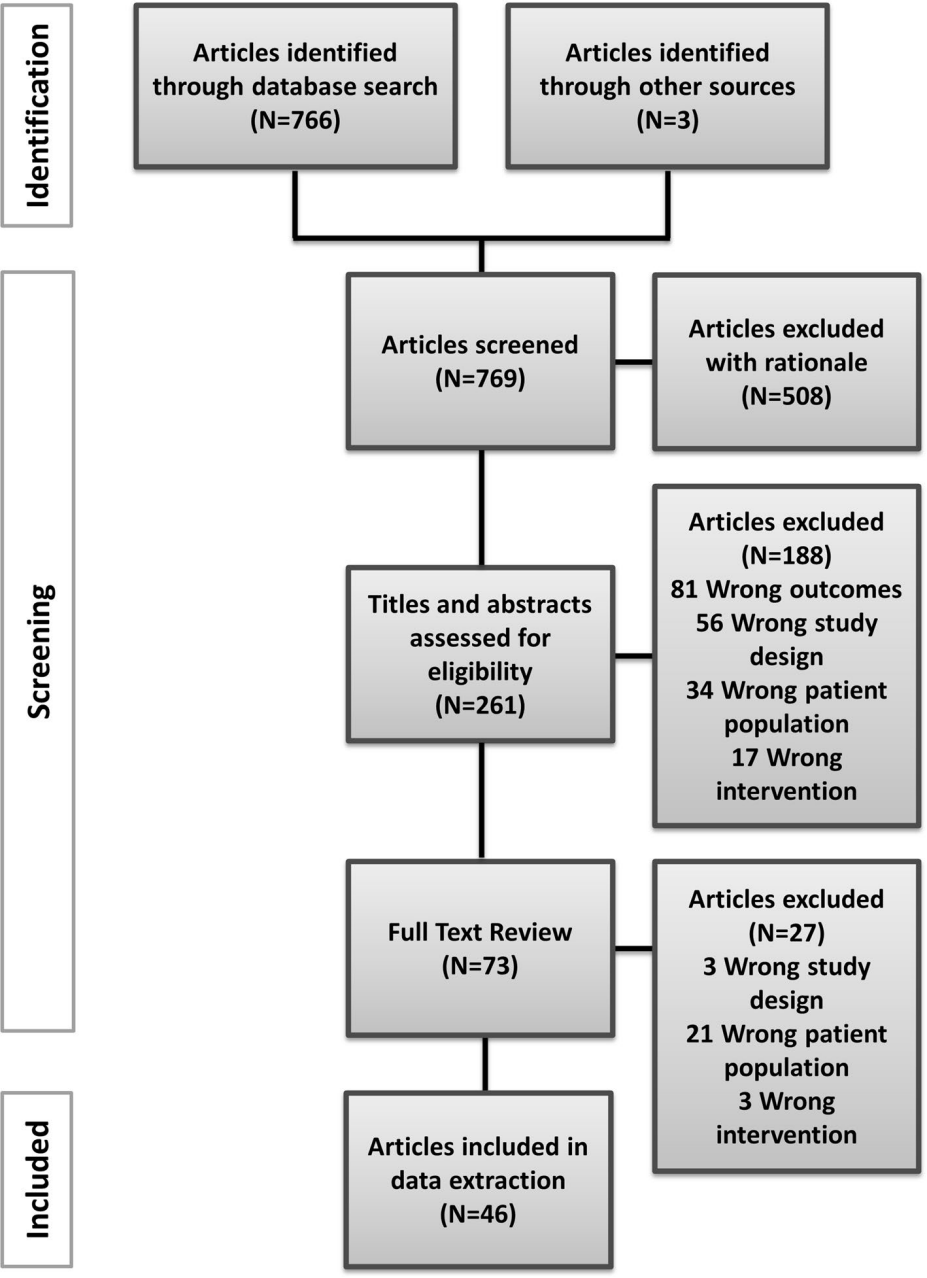
Prisma flow chart of literature review method

Sources were included if they reported on original studies that implemented transgender content within medical education and training, including residency programs. Additional inclusion criteria were reporting in English and date of publication in the last 10 years (2009–2019) which was updated in the revision process to 2021. Studies were excluded for the following reasons: (1) incorrect population (e.g., non-transgender, general LGBT+without discussing transgender content separately), (2) incorrect design (e.g., review, position statement, case report), (3) incorrect outcome (e.g., assessment of practitioners’ knowledge, experience and attitudes; perceived need for curricular reform rather than reporting on existing curricula), (4) incorrect intervention (e.g., not primarily on education, non-health education, nursing-, pharmacy-, or dental-education), (5) no abstract or article available. See Fig. 1 for the inclusion flowchart.

### Study selection

Consistent with the PRISMA statement (Page et al., 2021) studies were screened for eligibility in the following stages: title, title and abstract, full text (Fig. 1). After full-text reviews in stage three (*N* = 73), 27 articles were found not eligible based on incorrect study design, patient population, or intervention. The first three screening stages were completed by JvH, AK, and MM. The 46 remaining articles were discussed with TvdG and MM and were included for data extraction.

### Data extraction

The main data extracted from the articles are displayed in Tables 1 and 2. All data was extracted by JvH, AK, and MM and discussed with AH and TvdG. Basic characteristics of the training interventions were summarized in Table 1; these include location, year, number of students (total number, sometimes also including non-medical students/-residents), audience (e.g., students or residents), mandatory (required for graduation/embedded in core curriculum) or elective (with or without application process) intervention, frequency and duration of the course/training. “Not (further) specified” refers to information not *directly* stated in the article.

**Table 1:** Basic training characteristics

Reference	Location	Year	# of students	Audience	Mandatory or Elective	Frequency	Duration (h)
Altneu et al. (2020)	USA	NS	9	Undergraduate	Elective	Multi-Session	42 (1 Semester)
Arora et al. (2019)	Australia	NS	79	Undergraduate	NS	Single Session	1
Barrett et al. (2021)	USA	NS	31	Undergraduate & Residency	Elective	Single Session	2
Berenson et al. (2020)	USA	2018–2019	~ 178	Undergraduate	Mandatory	Multi-Session	2.5
Biro et al. (2021)	Canada	2016–2017	259	Undergraduate	Mandatory	Single Session	4
Bi et al. (2020)	USA	NS	89	Undergraduate	Mandatory	Single Session	2.5
Block et al. (2020)	USA	NS	NS	Undergraduate	NS	Single Session	NS
Braun et al. (2017)	USA	2015	62	Graduate	Elective	Multi-Session	10
Cherabie et al. (2018)	USA	NS	163	(Under)graduate^NS^ & Residency	Elective	Single Session	1
Click et al. (2020)	USA	2018	140	Undergraduate	Mandatory	Single Session	Half-day^NS^
Click et al. (2019)	USA	2018	140	Undergraduate	Mandatory	Single Session	Half-day^NS^
Congdon et al. (2021)	USA	2018	17	Undergraduate & Residency	Elective	Single Session	1
Cooper et al. (2018)	USA	NS	180	Undergraduate	NS	Single Session	1
Eriksson & Safer, 2016)	USA	NS	121	Undergraduate	Mandatory	Single Session	NS
Gibson et al. (2020)	USA	2017–2020	10–15	Undergraduate	Elective	Multi-session	4-years^NS^
Greene et al. (2017)	NS	NS	23	Residency	NS	Single Session	NS
Kidd et al. (2016)	NS	2015	34	Residency	Mandatory	Single Session	1.5
Levy et al. (2021)	USA	NS	51	Undergraduate	NS	Single Session	2
Mahabamunuge et al. (2021)	USA	2018–2020	186	(Under)graduate	Elective	Multi-Session	NS
Marshall et al. (2017)	NS	NS	163	Undergraduate	NS	Single Session	Half-day^NS^
McCave et al. (2019)	NS	2017–2018	494	Graduate	Mandatory	Single Session	NS
Minturn et al. (2021)	USA	2016–2017	49	Undergraduate	Elective	Multi-Session	10
Park & Safer, 2018)	USA	2014–2016	20	Undergraduate	Elective	NS	NS
Pathoulas et al. (2021)	USA	NS	54	Undergraduate	Elective	Single Session	1
Rosendale & Josephson, 2017)	USA	2015–2016	45	Residency	Mandatory	Multi-Session	6
Roth et al. (2021)	USA	2017–2019	~ 50	Residency	Mandatory	Multi-Session	NS
Roth et al. (2020)	NS	NS	146	Residency	Mandatory	Single Session	1–1.5
Ruud et al. (2021)	USA	NS	33	Residency	Elective	Single Session	2.25
Safer & Pearce, 2013)	USA	NS	180	Undergraduate	Mandatory	Single Session	NS
Salkind et al. (2019)	UK	2016–2019	NS	Undergraduate	Mandatory	Single Session	3
Sanchez et al. (2021)	USA	2017–2018	120	Undergraduate	NS	Single Session	2
Sawning et al. (2017)	USA	2014–2015	102	(Under)graduate	Elective	Multi-Session	11
Sequeira et al. (2012)	USA	2011–2012	NS	Undergraduate	Elective	Multi-Session	4
Stevenson et al. (2020)	USA	2016–2017	6	Residency	NS	Single Session	NS
Streed et al. (2019)	USA	2016–2018	833	Residency	NS	Single Session	1
Stumbar et al. (2021)	USA	2018–2019	113	Undergraduate	NS	Multi-Session	3.5
Taylor et al. (2018)	UK	2012–2015	350	Undergraduate	NS	Single Session	Half-day^NS^
Thompson et al. (2020)	USA	2018	127	Undergraduate	NS	Multi-Session	6.5
Ufomata et al. (2020)	USA	NS	153	Residency	Mandatory	Multi-Session	3
Underman et al. (2016)	NS	NS	179	Graduate	Mandatory	Single Session	2
Vance et al. (2017)	USA	2015–2016	20	Undergraduate	Elective	Multi-Session	7
Vance et al. (2020)	USA	NS	20	Residency, Graduate	Mandatory, Elective	Multi-Session	7
Vance et al. (2021)	USA	2018–2018	43	Residency, Graduate	Mandatory, Elective	Multi-Session	4.5
Vance et al. (2018)	USA	2016–2017	31	Undergraduate	Elective	Multi-Session	2
Wahlen et al. (2020)	Switzerland	2016	68	Graduate	Mandatory	Single Session	1
Ward-Gaines et al. (2021)	USA	NS	20	Residents	Mandatory	Single Session	2.5

NS Not further specified

**Table 2:** Pedagogical Features

Source	Delivery Format	Facilitator Role	Facilitator Qualifications	Framework	Transgender-Specific or LGBT+	Learning Objectives
Altneu et al. (2020)	Lecture, Videos, Small Group Discussions	Faculty, Health Professional/Other Content Expert(s), Student(s)/Resident(s)	Professional Expertise	AAMC Lesbian, Gay, Bisexual, and Transgender Healthcare, A Clinical Guide to Preventive, Primary, and Specialist Care(Eckstrand & Ehrenfeld, 2016)	General LGBT+	Awareness, Communication, Cultural Competency, Knowledge, Understanding
Arora et al. (2019)	Lecture	Professional/Other Content Expert(s), Transgender Person(s)	Lived Experience, Professional Expertise	(Coleman et al. (2012; Hembree et al. (2017; Telfer et al. (2018)	Transgender-specific	Attitudes, Awareness, Knowledge, Understanding
Barrett et al. (2021)	Lecture, Online Module, Role Play/Simulation, Videos	Faculty, Student(s)/Resident(s)	Professional Expertise	Lesbian, Gay, Bisexual, and Transgender Development of Clinical Skills Scale (LGBT-DOCSS) (Bidell, 2017)	General LGBT+	Attitudes, Awareness, Clinical Skills, Knowledge
Berenson et al. (2020)	Lecture, Panel, Small Group Discussion, Videos	Faculty, Health Professional/Other Content Expert(s), Student(s)/Resident(s)	Lived Experience, Professional Expertise	6-step Kern model (Thomas et al. (2015)	Transgender-specific	Awareness, Communication, Cultural Competency, Knowledge
Biro et al. (2021)	Live Interview, Role Play/Simulation, Small Group Discussions	Faculty, Health Professional/Other Content Expert(s), Student(s)/Resident(s)	Lived Experience, Professional Expertise	NS	General LGBT+	Awareness, Clinical Skills, Communication
Bi et al. (2020)	Lecture, Live Interview, Videos	Health Professional/Other Content Expert(s), Student(s)/Resident(s), Transgender Person(s)	Lived Experience, Professional Expertise	Intersectionality	General LGBT+	Awareness, Communications, Cultural Competency, Knowledge
Block et al. (2020)	Lecture, Small Group Discussions	Faculty, Health Professional/Other Content Expert(s), Student(s)/Resident(s)	Professional Expertise	NS	General LGBT+	Attitudes, Clinical Skills, Knowledge, Understanding
Braun et al. (2017)	Lecture	Faculty, Health Professional/Other Content Expert(s), Student(s)/Resident(s), Transgender Person(s)	Lived Experience, Professional Expertise	Genderism and Transphobia Scale (Nagoshi et al., 2008)	Transgender-specific	Advocacy, Awareness, Knowledge, Understanding
Cherabie et al. (2018)	Lecture	Transgender Person(s)	Lived Experience	NS	Transgender-specific	Attitudes, Knowledge, Understanding
Click et al. (2020)	Lecture, Live Interview, Small Group Discussions	Faculty, Students, Transgender Person(s)	Lived Experience, Professional Expertise	AAMC	Transgender-specific	Attitudes, Awareness, Knowledge, Communication
Click et al. (2019)	Lecture, Live Interview, Small Group Discussions	Faculty, Student(s), Transgender Person(s)	Lived Experience, Professional Expertise	AAMC	Transgender-specific	Attitudes, Awareness, Knowledge, Communication
Congdon et al. (2021)	Lecture	Student(s)/Resident(s)	Professional Expertise	(Dubin et al. (2018)	Transgender-specific	Knowledge
Cooper et al. (2018)	Lecture	Health Professional/Other Content Expert(s)	Professional Expertise	AAMC	General LGBT+	Advocacy, Awareness, Knowledge, Understanding
Eriksson & Safer, 2016)	Lecture	NS	NS	NS	Transgender-specific	Attitudes, Awareness, Knowledge
Gibson et al. (2020)	Lecture, Online Module, Panel Discussion, Community Service, Mentorship	Health Professional/Other Content Expert(s), Student(s)/Resident(s), Transgender Person(s)	Lived Experience, Professional Expertise	AAMC	General LGBT+	Advocacy, Awareness, Clinical Skills, Communication, Knowledge, Understanding
Greene et al. (2017)	Role Play/Simulation	Faculty, Transgender Person(s)	Lived Experience	NS	Transgender-specific	Communication
Kidd et al. (2016)	Lecture, Role Play/Simulation	Health Professional/Other Content Expert(s)	Consulting LGBT+Organization, Professional Expertise	NS	Transgender-specific	Attitudes, Awareness, Communication, Understanding
Levy et al. (2021)	Case Based Learning, Small Group Discussions	LGBT+-identifying person(s), Transgender Person(s)	Lived Experience	NS	General LGBT+	Attitudes, Cultural Competency, Knowledge
Mahabamunuge et al. (2021)	Lecture	Health Professional/Other Content Expert(s), LGBT+-identifying person(s)^NS^	Lived Experience, Professional Expertise	NS	Dedicated transgender-focus within predominantly LGBT+curriculum	Attitudes
Marshall et al. (2017)	Lecture, Live Interview, Videos, Panel Discussion	Faculty, Transgender Person(s)	Lived Experience, Professional Expertise	AAMC	Transgender-specific	Awareness, Knowledge, Communication, Understanding
McCave et al. (2019)	Lecture, Videos, Live Interview, Small Group Discussions, Role Play	Faculty, Transgender Person(s), Student(s)/Resident(s)	Lived Experience, Professional Expertise	NS	Transgender-specific	Clinical Skills, Advocacy, Knowledge
Minturn et al. (2021)	Lecture, Role Play/Simulation, Case Based Learning, Panel Discussion	Faculty, Transgender Person(s), LGBT+-identifying person(s), Student(s)/Resident(s)	Lived Experience, Professional Expertise, Consulting transgender patients/community members, Consulting LGBT+Organization	NS	General LGBT+	Attitudes, Understanding, Cultural Competency, Knowledge
Park & Safer, 2018)	Clinical Rotations, Lecture	NS	NS	AAMC approach with evidence-based, transgender specific medical education	Transgender-specific	Attitudes, Awareness, Communication
Pathoulas et al. (2021)	Lecture, Live Interview	NS	NS	NS	Transgender specific	Clinical Skills, Knowledge
Rosendale & Josephson, 2017)	Lecture	NS	NS	NS	Dedicated transgender-focus within predominantly LGBT+curriculum	Clinical Skills, Communication, Understanding, Cultural Humility
Roth et al. (2021)	Lecture, Small Group Discussions, Case Based Learning	Health Professional/Other Content Expert, Student(s)/Resident(s)	NS	AAMC, AAP	General LGBT+	Advocacy, Communication, Awareness, Understanding, Knowledge, Clinical Skills
Roth et al. (2020)	Case Based Learning	Faculty, Student(s)/Resident(s)	NS	AAMC	General LGBT+	Knowledge, Clinical Skills
Ruud et al. (2021)	Role Play/Simulation	Health Professional/Other Content Expert(s), Transgender Person(s)	Lived Experience, Professional Expertise, Consulting transgender patients/community members	NS	Transgender and non-binary/gender non-conforming	Attitudes, Knowledge, Clinical Skills
Safer and Pearce (2013)	Lecture	NS	NS	NS	Transgender-specific	Attitudes
Salkind et al. (2019)	Lecture, Live Interview, Role Play/Simulation	Health Professional/Other Content Expert(s)*,* LGBT+-identifying person(s), Transgender Person(s)	Consulting transgender patients/community members, Lived Experience	NS	Balanced (transgender/LGBT+)	Advocacy, Knowledge, Communication, Understanding
Sanchez et al. (2021)	Lecture, Reflective writing exercise, Case Based Learning	Faculty, Health Professionals/Other Content Expert(s), Student(s)/Resident(s)	Lived Experience, Professional Expertise	AAMC	General LGBT+	Knowledge
Sawning et al. (2017)	Panel Sessions^NS^	Health Professional/Other Content Expert(s), LGBT+-identifying person(s)^NS^	Lived Experience^NS^, Professional Expertise	AAMC	Dedicated transgender-focus within predominantly LGBT+curriculum	Attitudes, Knowledge, Cultural Competency
Sequeira et al. (2012)	Lecture, Role Play/Simulation	NS	NS	NS	Balanced (transgender/LGBT+)	Knowledge, Communication, Understanding, Awareness
Stevenson et al. (2020)	Role Play/Simulation, Case Based Learning	Faculty, Health Professional/Other Content Expert(s), LGBT+-identifying person(s)	Professional Expertise	NS	Transgender-specific	Knowledge, Clinical Skills, Communication
Streed et al. (2019)	Online Module	NS	NS	AAMC, DHHS & CDC and unnamed “educational strategy for addressing SGM health disparities”	General LGBT+	Clinical Skills, Knowledge, Understanding
Stumbar et al. (2021)	Lecture, Role Play/Simulation	Faculty, Health Professional/Other Content Expert(s), Transgender Person(s), Student(s)/Resident(s)	Consulting Transgender Patients/Community Members, Professional Expertise	5P’s model (Reno et al. (2019)	Transgender-specific	Knowledge, Clinical Skills
Taylor et al. (2018)	Lecture, Role Play/Simulation	Student(s)/Resident(s), LGBT+-identifying person(s)^NS^	Lived Experience^NS^	NS	General LGBT+	NS
Thompson et al. (2020)	Lecture, Videos, Case Based Learning, Role Play/Simulation, Panel Discussion	Faculty, Health Professional/Other Content Expert(s), Transgender Person(s)	Lived Experience, Professional Expertise	NS	Transgender and non-binary/gender non-conforming	Knowledge, Clinical Skills, Attitudes, Competency
Ufomata et al. (2020)	Case Based Learning	Faculty, Health Professional/Other Content Expert(s)	Nonexpert faculty	AAMC	General LGBT+	Cultural Competency, Understanding, Knowledge, Attitudes
Underman et al. (2016)	Live Interview	Faculty, Health Professional/Other Content Expert(s)	Consulting Transgender Patients/Community Members, Professional Expertise	AAMC	Transgender-specific	Communication
Vance et al. (2017)	Clinical Rotations, Online Module	Faculty	Professional Expertise	NS	Transgender-specific	Knowledge, Understanding
Vance et al. (2020)	Online Module, Videos, Clinical Rotations	Health Professional/Other Content Expert(s)	Professional Expertise	NS	Transgender-specific	Knowledge, Awareness
Vance et al. (2021)	Online Module, Video Interview, Role Play/Simulation	Faculty, Health Professional/Other Content Expert(s), Transgender Person(s)	Lived Experience, Professional Expertise	NS	Transgender-specific	Clinical Skills, Communication
Vance et al. (2018)	Clinical Rotations, Online Module	Faculty	Professional Expertise	NS	Transgender-specific	Clinical Skills, Knowledge
Wahlen et al. (2020)	Lecture	Professional/Other Content Expert(s)	Professional Expertise	NS	General LGBT+	Knowledge, Attitudes
Ward-Gaines et al., 2021)	Role Play/Simulation	Faculty, Professional/Other Content Expert(s)	Lived Experience^NS^, Professional Expertise	NS	Dedicated transgender-focus within health equity curriculum	Awareness, Understanding, Clinical Skills

*AAMC* The Association of American Medical Colleges (2014) “Implementing Curricular and Institutional Climate Changes to Improve Health Care for Individuals Who Are LGBT, Gender Nonconforming (GNC), or Born with Differences of Sex Development (DSD).”, *AAP* American Academy of Pediatrics: Levine, D. A., Braverman, P. K., Adelman, W. P., Breuner, C. C., Marcell, A. V., Murray, P. J., & O’Brien, R. F. (2013). Office-based care for lesbian, gay, bisexual, transgender, and questioning youth. *Pediatrics*, *132*(1), e297-e313, *CDC* (Department of Health and Human Services (DHHS) and Centers for Disease Control and Prevention (CDC), 2014), *SGM* sex and gender minorities, *LGBT* + Lesbian, Gay, Bisexual and Transgender [and other non-hegemonic gender identities and sexual orientations], NS Not further specified

Table 2 expands upon Table 1 with additional details pertaining to features that were considered distinctive of medical training in the area of transgender health. The indicator Delivery Format was chosen to get a sense of *how* the training interventions were being delivered and how much direct contact (e.g., in-person vs. online classes) the intervention provided. Facilitator Role helped to understand who was involved in developing, delivering and evaluating the training (e.g., faculty, medical professionals, transgender individuals). Similarly, Facilitator Qualifications captured what expertise and/or experience qualified them to teach the material (e.g., their epistemic and authoritative legitimacy). Data for the next indicator was collected to note whether the intervention was informed by an existing/acknowledged Framework (e.g., AAMC). Data for the Transgender-Specific/LGBT+indicator were extracted for a number of reasons: (a) to understand whether transgender objectives were considered unique from LGBT+topics or grouped together, (b) to get a sense of whether gender and sexuality were addressed independently or conflated as a homogenous concept, and (c) to further situate the learning objectives within the appropriate context. Lastly, information about the knowledge, attitudes, skills, and consciousness (specifically related to transgender health) that students and/or residents were expected to acquire through the training was collected for the Learning Objectives indicator.

In addition to extracting basic training characteristics and pedagogical features, we also extracted data for barriers and challenges encountered while implementing training interventions as well as strategies that facilitated and strengthened training interventions. This data was collected, summarized and thematically analyzed to understand how training interventions are being delivered and what factors are influencing their implementation. By highlighting these experiences, we aim to support curriculum designers and instructors to successfully plan and deliver training that will advance transgender health equity in medical curricula.

## Results

### Study characteristics

Most studies were conducted in the USA (*N* = 13) and conducted between 2011 and 2020 (Table 1). The number of students in each class ranged from 6 to 833 with a mean of 109 students, mostly of undergraduate level (65%). The majority of articles described single-session interventions (*N* = 28) with a duration range of one hour to a half-day.

Pedagogical features of the interventions are displayed in Table 2. Eleven studies noted the exclusive use of lectures as the intervention, whereas most studies (*N* = 29) reported multimodal initiatives and included a combination of delivery formats. Most studies (*N* = 34) had multiple types of developers/facilitators including: faculty, health professionals/other content experts, students/residents, transgender individuals, and/or LGBT+-identifying individuals. Relatedly, expertise and/or experience qualifying the facilitators included (a combination of) the following qualifications: lived experience, professional expertise, consulting LGBT+organization, and/or consulting transgender patients/community members. Twenty-two articles included facilitators with lived experience (e.g., transgender and/or LGBT+individuals). Most articles (*N* = 25) did not mention using a framework either to guide development of curricula or to situate their work. Most studies that did used the AAMC (Hollenbach et al., 2014) framework (*N* = 14). After screening for articles with a significant focus on transgender health, twenty-two of the included sources focused specifically on transgender content, two also on non-binary/gender non-conforming content, sixteen on general LGBT+content, five on both, and one on health equity in general (e.g., including ethnicity). Lastly, most studies (*N* = 39) mentioned a combination of learning objectives, including advocacy, awareness, knowledge, understanding, attitudes, communication, cultural competency and/or clinical skills. Closer analysis of learning objectives revealed that learning goals generally lacked completeness, in particular related to content about clinical communication with transgender patients, critical reflexivity on student’s individual views on sex and gender, understanding patients’ identities and experiences through an intersectionality lens, and providing good care to transgender patients outside of specialized transition care.

### Barriers and facilitators meta-themes

Thematic analysis highlighted four areas in which both barriers and facilitators in implementing training related to transgender health commonly manifested: training intervention (e.g., factors related to the course or curricular design), facilitation (e.g., factors present among instructors’ and guest speakers), audience (e.g., factors present among the medical students/residents), and institutional/contextual (e.g., factors shaped by broader organizational, social, cultural and/or political dynamics). All areas of barriers and facilitators are described and specified in the Supplementary Materials.

### Barriers and challenges encountered when implementing training interventions

The most commonly reported barrier related to the training interventions was lack of appropriate educational materials (Park & Safer, 2018; Rosendale & Josephson, 2017; Vance et al., 2020, 2021). Facilitation-related barriers primarily centered around the lack of expertise or experience among faculty or instructors to facilitate the training (Berenson et al., 2020; Biro et al., 2021; Block et al., 2020; Click et al., 2019, 2020; Greene et al., 2017; Marshall et al., 2017; McCave et al., 2019; Park & Safer, 2018; Rosendale & Josephson, 2017; Sawning et al., 2017; Sequeira et al., 2012; Stumbar et al., 2021; Thompson et al., 2020; Ufomata et al., 2020; Vance et al., 2018) and availability of transgender simulation patients (Berenson et al., 2020; Biro et al., 2021; Block et al., 2020; Click et al., 2019, 2020; Greene et al., 2017; Marshall et al., 2017; McCave et al., 2019; Park & Safer, 2018; Rosendale & Josephson, 2017; Sawning et al., 2017; Sequeira et al., 2012; Stumbar et al., 2021; Thompson et al., 2020; Ufomata et al., 2020; Vance et al., 2018). Low participation rates among certain subgroups (e.g., male and cisgender people) was the most reported barrier related to the audience (Braun et al., 2017; Minturn et al., 2021; Pathoulas et al., 2021). Institutional/contextual barriers included time constraints in the majority of sources and costs associated with consultation, curriculum development, as well as recruiting and fairly compensating transgender simulation patients/guest speakers (Altneu et al., 2020; Bi et al., 2020; Biro et al., 2021; Braun et al., 2017; Click et al., 2019; Cooper et al., 2018; Kidd et al., 2016; Marshall et al., 2017; Minturn et al., 2021; Rosendale & Josephson, 2017; Roth et al., 2020; Salkind et al., 2019; Sawning et al., 2017; Stumbar et al., 2021; Ufomata et al., 2020; Underman et al., 2016; Vance et al., 2020; Ward-Gaines et al., 2021). Kidd and colleagues critiqued the limited effectiveness of ‘one-shot’ educational interventions, on which much of residency education relies, [which] may not necessarily result in sustained improvements” (2016, p. 4). Furthermore, Cooper et al. (2018) and Barrett et al. (2021) noted shortcomings related to time constraints, which made it impossible to adequately address the unique needs of all subgroups within the LGBT+umbrella, including but not limited to individuals with a trans-masculine identity and those with a trans-feminine identity.

### Strategies to facilitate and strengthen training interventions

To address many of the common barriers identified above, authors highlighted strategies that helped facilitate and strengthened their training interventions. Use of adaptable or flexible formats such as asynchronous online modules and pre-existing video resources were often mentioned as beneficial strategies to training implementation (Altneu et al., 2020; Barrett et al., 2021; Bi et al., 2020; Braun et al., 2017; Kidd et al., 2016; Pathoulas et al., 2021; Streed et al., 2019; Ufomata et al., 2020; Vance et al., 2017, 2018, 2020; Ward-Gaines et al., 2021). The sources also featured a variety of instructional methods to engage students in active learning. The most favored approach to training medical students and residents in transgender health was the use of interactive modalities such as case-based formats, clinical scenarios, mock patient encounters, role-play and/or live interviews that provided students with opportunities to practice interviewing skills and conducting a history with a transgender patient (Altneu et al., 2020; Barrett et al., 2021; Berenson et al., 2020; Bi et al., 2020; Biro et al., 2021; Click et al., 2019; Greene et al., 2017; Kidd et al., 2016; McCave et al., 2019; Park & Safer, 2018; Salkind et al., 2019; Sequeira et al., 2012; Streed et al., 2019; Stumbar et al., 2021; Taylor et al., 2018; Underman et al., 2016; Vance et al., 2017, 2018, 2021; Ward-Gaines et al., 2021). Authors also suggested the use of group discussions and debriefing sessions to compliment and build upon interactive activities, which presented opportunities to provide guidance, answer questions and work through any tension or discomfort (Kidd et al., 2016; Salkind et al., 2019; Sequeira et al., 2012; Underman et al., 2016).

The sources mentioned several strengths of their training interventions associated with facilitation. Many of the sources emphasized the importance of drawing on the expertise and lived experience of transgender patients/simulation patients to help support curriculum development and delivery. Authors cited multiple benefits such as constructing realistic clinical scenarios and providing a more authentic and personal encounter for medical students/residents, which contribute to enhanced long-term retention of knowledge (Arora et al., 2019; Berenson et al., 2020; Bi et al., 2020; Biro et al., 2021; Marshall et al., 2017; McCave et al., 2019; Salkind et al., 2019; Stumbar et al., 2021; Underman et al., 2016; Vance et al., 2021). Furthermore, the involvement of students, especially LGBT+students, in curriculum development and delivery was identified as an asset (Braun et al., 2017; Click et al., 2019; Taylor et al., 2018).

Other facilitators were commonly identified at the audience level about which most authors referred to the advantage of students’/residents’ pre-existing awareness, knowledge, positive attitudes, interest, perceived significance, prior professional and/or lived experience that was a foundation for further learning on the topic of transgender health (Berenson et al., 2020; Click et al., 2019, 2020; Cooper et al., 2018; Eriksson & Safer, 2016; Gibson et al., 2020; Levy et al., 2021; Marshall et al., 2017; Park & Safer, 2018; Safer & Pearce, 2013; Sawning et al., 2017; Taylor et al., 2018; Underman et al., 2016; Vance et al., 2017). In some cases, this was linked to “the fact that [the training] was elective and might have attracted a sample of students who were already interested and informed on the topic” (Eriksson & Safer, 2016, p. 840). In other cases, authors pointed to the value of prior exposure to gender and sexuality or transgender-specific content integrated in the medical curriculum preceding the training intervention (Berenson et al., 2020; Braun et al., 2017; Marshall et al., 2017; Park & Safer, 2018; Safer & Pearce, 2013; Streed et al., 2019). Others attributed it to newer generations of medical students being inherently more receptive to these topics as a result of the increased visibility of LGBT+people in society (Cooper et al., 2018; Eriksson & Safer, 2016; Marshall et al., 2017; Safer & Pearce, 2013). On the other hand, Altneu et al. (2020) noted that the deliberate inclusion of a diverse participant group across disciplinary backgrounds and with varying levels of prior exposure to the topic enriches the learning experience.

Finally, institutional and contextual factors were also assessed as helping to facilitate implementation of training in transgender health in medical schools. As noted above, students’ prior exposure to related educational content was supported through vertically and horizontally scaffolding learning throughout medical curricula (Park & Safer, 2018). This scaffolding required institutional/faculty buy-in and dedication of time and resources (Gibson et al., 2020; Ruud et al., 2021; Vance et al., 2017, 2018, 2020, 2021). Support across medical schools and academic institutions was in turn shaped by organizational culture and the broader sociopolitical climate (Biro et al., 2021; Eriksson & Safer, 2016; Gibson et al., 2020; Rosendale & Josephson, 2017; Safer & Pearce, 2013; Sawning et al., 2017).

## Discussion

The aim of this theory-guided systematic review was to (1) provide an overview of key characteristics of training initiatives and pedagogical features, (2) analyze barriers and facilitators to implementing this training in medical education, and (3) interpret our findings through the lens of queer theory. We found that most training consisted of single-session interventions, were multimodal, and the number of trainings covering general LGBT+—and specific transgender-content were equal. Furthermore, learning goals and pedagogical strategies generally lacked completeness, in particular related to content about clinical communication with transgender patients, critical reflexivity on student’s individual views on sex and gender, understanding patients’ identities and experiences through an intersectionality lens, and providing good care to transgender patients outside of specialized transition care.

Barriers to training implementation included lack of educational materials, lack of experienced staff or (simulation) staff with transgender lived experience, lack of ties to the transgender community, and time and costs constraints. In line with previous research (Pratt-Chapman, 2020) and widely-recognized implementation standards (Hollenbach et al., 2014), facilitators included using flexible formats, scaffolding learning throughout the curriculum, employing co-creative and multi-disciplinary approaches to development and delivery of educational content, and engaging learners in skills-based training.

### Queering medical knowledge, medical dualism, and the pathologization of transgender bodies

In line with previous findings, we found that across all levels of medical education varying initiatives to address transgender health issues are being implemented and evaluated (Nolan et al., 2020; Pratt-Chapman, 2020). However, such initiatives are often isolated or not structurally embedded in the curriculum, do not always reach all students, and do not always have access to the pedagogical conditions (e.g., experienced and knowledgeable staff, curriculum time and financial and material resources) necessary to optimize teaching and learning about transgender health. Educators also urge for more emphasis on the incorporation of complexity in sessions about transgender health, for instance by exploring identity factors that intersect with transgender identity, such as sexuality, ethnicity/race, class and age in teaching material (Barrett et al., 2021; Bi et al., 2020; Biro et al., 2021; Levy et al., 2021; Muntinga et al., 2020). And while we found that involvement of transgender perspectives in developing and delivering content was beneficial for student learning and part of necessary advocacy work, some authors warn of overburdening people with lived experiences (including transgender students), in particular when experiential knowledge cannot be properly compensated (Bi et al., 2020; Biro et al., 2021). Finally, there is concern about how to optimize learner gain; currently, no conclusive evidence exists as to which timing, modality, and method of implementation effectively contributes to student learning and clinical outcomes (Altneu et al., 2020), while the everyday conditions of graduate, undergraduate and residency programs hinder initial implementation of curricula (Donovan et al., 2021; Moll et al., 2021). All of this suggests that currently, medical curricula are lagging to take a key role as intervention sites for reducing and eliminating transgender health inequities (Giffort & Underman, 2016; Pregnall et al., 2021).

Following queer theorists and building on the work of critical diversity scholars, we argue that this lagging can partly be located in the foundational fabrics of the biomedical paradigm. As an organizational principle of medical practice, western biomedical dualism relies on the use of often binary categories to classify health and disease and predict disease patterns and trends. While this approach has proven valuable and even unavoidable at the population level – for instance to write health policy and implement public health programs – at the patient level it translates to a reductionist application of information derived from statistical averages (Epstein, 2008). As such, it erases the complexity that is inherent to individual human bodies, behaviors and experiences. In addition, as Foucault has outlined in his book ‘The birth of the clinic’, while biomedical knowledge claims scientific objectivity, universality and neutrality (see also Beagan, 2000), the clinical gaze assigns social and cultural meaning to what it observes and has discursive dominance over. In a context in which biomedical knowledge has historically gained authority to mandate moral and physical standards of being and acting, biomedicine has, as William Spurlin writes, assumed “a normative posture” (Foucault, 2012; Spurlin, 2019). In other words, medical knowledge has the sociopolitical power to dictate and regulate what is ‘normal’ and ‘pathological’ (Gisondi & Bigham, 2021). According to Foucault, this includes the polar structuring of bodies and behaviors into gendered and sexualized categories, where ‘normal’ links the moral with the physical (Spurlin, 2019). As such, medical dualism sustains a classification system where the healthy norm is the socially conforming cisgender and heterosexual patient, while everyone else is ‘the pathological Other’ (Murphy, 2016; Spurlin, 2019).

In medicine and medical education, binary systems of knowledge about bodies clearly show up in the shape of hetero- and cisgender-centrist environments (Hil Malatino, 2020; Murphy, 2016). In such gender normative environments, transgender identities are organized as distinctively different from cisgender identities in course designs and materials, and as embodying socio-physical realities that provide a diagnostic cue (Gisondi & Bigham, 2021; Muntinga et al., 2020; Robertson, 2017). The hierarchy inherent to this system is reflected in the extracurricular nature of some training initiatives, and in the relative lack of learning goals that focus on providing care for transgender patients in general care settings. Educational interventions that address transgender health content separately from cisgender content cause people with more complex gender realities to be overlooked as ‘regular’ patients, while at the same time they are brought into consciousness only in the context of deviance, inadvertently keeping pathologization of transgender bodies in place (Hil Malatino, 2020). In addition, separating transgender health issues from cisgender health issues not only confirms normative gender ideologies that classify transgender people as ‘unhealthy Others’, it also reinforces the biased notion that cisgender experiences of gender are natural, fixed and uncomplicated (Jacobson & Joel, 2018). Hence, the binary organization of transgender-related content in medical curricula of gender might impact cisgender patients as well.

We argue, therefore, that the the conditions for trans-inclusive medical education require a critical investigation of not just the medical category 'transgender', but also of the medical categories 'male' and ' female'. Throughout the curriculum, students should be encouraged to think of gender and sex as a universally lived experience, where cisgender and transgender (and intersex) patients have diversity and complexity. Implementing such pedagogies in the formal, informal and hidden curriculum have the potential to remove gender bias from everyday clinical care interactions, and could challenge, for instance, neurosexist practices in sex difference research (Broussard et al., 2018; Fine, 2013). Moreover, they have a force in demystifying transness, which further aids in de-pathologization of transgender lives and bodies.

### Journey toward trans-inclusive medical education

Following the ‘the three fixes’ model (Verdonk & Janczukowicz, 2018), we imagine the journey toward trans-inclusive medical education, then, first as *fixing medical knowledge* about sex, gender and transgender bodies. In their 2019 essay, Chris Barcelos argues that a collective shift in consciousness might be best achieved by bringing transfeminist pedagogies into the health classroom (Barcelos, 2019). Briefly summarized, such a perspective complicates widely-held assumptions about the relationship between the genotypical and phenotypical body. For instance, in a curricula guided by a transfeminist framework, students are encouraged to think of pregnant or menstruating people not only as female, to consider gender diverse patients across all their sexual and romantic orientations, to be aware of reproductive pathology such as endometriosis or ovarian cancer in males, to understand female identities beyond female genitalia, or to avoid automatically linking health complaints of transgender patients with GHT use, gender dysphoria, or past surgeries. Trans-inclusive curricula should be explicitly intersectional – they should consider the experiences of patients to be shaped by other layered aspects of identity such as racialized identity/ethnicity, culture, age, class and [dis]ability (Aizura et al., 2020; Muntinga et al., 2020). They should also be transdisciplinary— students should be provided with the latest insights from the humanities, social sciences, and biomedical sciences regarding explanatory models for gender identity (including cisgender identity) and sex differentiation alongside (appropriately reimbursed) expertise that transcends traditional boundaries of disciplines and incorporates sources of knowledge often silenced or discredited by western academia (e.g., lived experience). At the level of *fixing the numbers*, we believe that trans-inclusivity should also mean hiring (and adequately compensating) educators and didactic specialists from transgender communities (including doctors) to speak to transgender health experiences. Finally, *fixing the institution* implies creating the pedagogical, policy, material, financial and organizational-cultural conditions to establish learning environments in which the complexity of constellations of gender and sexuality can be collectively reflected on, investigated and understood. Because barriers to teaching transgender health in medical education are rooted in the binary gendered ideology of medical thought and thus medical education, trans-inclusive medical education will not be gained without broader curricular and institutional climate change (Hollenbach et al., 2014). This ‘thinking with trans’ in medical education would require a comprehensive approach, in which gender complexity is structurally addressed at all levels of the institution and “outside of trans as subject position” (Aizura et al., 2020). Moreover, this necessitates eliminating hostile environments and fostering safe learning spaces in which students and teachers with lived experience and experiential knowledge feel safe to contribute to the conversations.

### Strengths and limitations

This theory-guided review is one of the first studies to provide an overview of and critically analyze training initiatives on the topic of transgender healthcare in medical education. To deepen our understanding of how to move forward with trans health in medical education, we applied queer theory; although sparsely used within the medical sciences, a theory driven design is novel when used in the context of evidence synthesis, and we believe our analysis shows how it can lead to novel insights about ways to curriculize health issues of minoritized and equity-seeking populations. Furthermore, this review follows the PRISMA guidelines (Page et al., 2021), increasing replicability. Finally and importantly, the diversity of sex, gender identity, and sexual orientation among the authors aids in optimizing this study’s credibility.

However, the results of this review must be viewed in light of several limitations. First, only sources published in English were included, which limits this study’s representation of and relevance to non-Western cultural contexts. Moreover, all except three selected articles that specified the study’s location were conducted in the USA or UK. Second, some of the barriers/facilitators identified are not unique to transgender health education. Third, this review was originally set out to capture promising practices in transgender and differences in sex development pedagogy; however, current literature does not provide sufficient detail on these topics. Last, we did not conduct a comprehensive critical appraisal of the quality of literature.

### Conclusion

The aim of this review was to assess the current state of training related to transgender health in medical student curricula and residency programs, extract barriers and facilitators to implementing training interventions, and to contextualize our findings using queer theory. We conclude that, although we were able to identify promising practices, there are still systemic barriers to structurally and sustainably implement transgender health content into medical education. These barriers might be rooted in the cisgender normativity of medical knowledge, which classifies transgender bodies as inherently pathological. Optimizing medical education as a site of intervention towards transgender health equity requires pedagogical approaches that critically interrogate and complicate sex and gender categories and their relation to health experiences. Such approaches to training future doctors might not only benefit transgender patients, but also cisgender patients.

## Positionality statement

We are a group of individuals of different genders and sexualities. JvH, AH, TvdG and MM are white and Dutch, AK is a white settler on unceded Coast Salish territories. At the time of publication, JvH is a physician and researcher at Amsterdam University Medical Center’s Center of Expertise on Gender Dysphoria, AK is a PhD Candidate at Simon Fraser University’s Faculty of Health Sciences, TvdG is a resident in psychiatry and sexual medicine, and assistant professor at Amsterdam University Medical Center’s Center of Expertise on Gender Dysphoria (dept. of Plastic, Reconstructive and Hand Surgery), AH is a practicing physician at the Centre for Sexual Health of the GGD Amsterdam, and MM works as an assistant professor at Amsterdam University Medical Center’s department of Ethics, Law and Humanities.

## Supplementary Information

Below is the link to the electronic supplementary material.

Supplementary file1 (DOCX 239 kb)
