# Supplementary material for: Transgender health content in medical education: a theory-guided systematic review of current training practices and implementation barriers & facilitators
Source: Adv Health Sci Educ Theory Pract. 2022 Apr 12;27(3):817–46. doi: 10.1007/s10459-022-10112-y (PMC9374605; doi:10.1007/s10459-022-10112-y)
Supplement: Supplementary file 1 — Supplementary file1 (DOCX 239 kb) [file 10459_2022_10112_MOESM1_ESM.docx]

**Supplementary Materials**

**Barriers and facilitators meta-themes**

Barriers

*Training Intervention:*

- Limited exposure to transgender people/patients
  - (Park and Safer, 2018; Vance et al., 2018)
- Limited capacity of the elective/ educational materials
  - (Rosendale and Josephson, 2017; Park and Safer, 2018; Vance et al., 2020; Vance et al., 2021)
- Elective intervention/ not embedded in core curriculum
  - (Park and Safer, 2018; Sequeira et al., 2012)
- Challenges with capturing clinical scenarios through online modules
  - (Eriksson and Safer, 2016)
- Lack of representation of diversity
  - (Barrett et al., 2021)

*Facilitation:*

- Lack of experienced staff
  - (Click et al., 2019; Marshall et al., 2017; Rosendale and Josephson, 2017; Sawning et al., 2017; Biro et al., 2021; Click et al., 2020; Thompson et al., 2020; Ufomata et al., 2020)
- Lack of transgender or non-binary staff: projects planned and led by primarily cisgender people.
  - (Ruud et al., 2021; Thompson et al., 2020)
- Discomfort with the topic
  - (Sawning et al., 2017; Altneu et al., 2020)
- Availability of transgender simulation patients
  - (Greene et al., 2017; Marshall et al., 2017; Park and Safer, 2018; Sequeira et al., 2012; Vance et al., 2018; Berenson et al., 2020; Biro et al., 2021; Block et al., 2020; McCave et al., 2019; Stumbar et al., 2021)

*Audience:*

- Low lecture attendance
  - (Salkind et al., 2019; Eriksson and Safer, 2016)
- Discomfort with the topic
  - (Sawning et al., 2017)
- Low participation rates among certain subgroups: likely that students entering the course have more prior experience with and positive attitudes towards LGBTQI+ individuals
  - (Braun et al., 2017; Minturn et al., 2021; Pathoulas et al., 2021)
- Late exposure to topic: already developed skills/ practices that might not be most fitting to the LGBTQI+ patient context
  - (Roth et al., 2020)
- Large group sizes
  - (Roth et al., 2020)
- Increased workload for students
  - (Sawning et al., 2017)
- Limited background knowledge or exposure
  - (Altneu et al., 2020)

*Institutional/ Contextual:*

- Financial constraints
  - (Braun et al., 2017; Underman et al., 2016; Minturn et al., 2021; Stumbar et al., 2021; Ufomata et al., 2020)
- Time constraints
  - (Kidd et al., 2016; Click et al., 2019; Cooper et al., 2018; Marshall et al., 2017; Rosendale and Josephson, 2017; Salkind et al., 2019; Sawning et al., 2017; Roth et al., 2020; Altneu et al., 2020; Biro et al., 2021; Bi et al., 2020; Vance et al., 2020; Ward-Gaines et al., 2021)
- Concerns about curricular overcrowding
  - (Sawning et al., 2017)
- Rarity of transgender (youth) clinical programs itself
  - (Vance et al., 2017)
- COVID-19: Inability to interact in person
  - (Barrett et al., 2021; Levy et al., 2021)
- Lack of improvement regarding this topic on an institutional level (e.g., support staff, nursing staff, psychologists etc.)
  - (Thompson et al., 2020)

Facilitators

*Training Intervention:*

- Training during lunchtime hours
  - (Braun et al., 2017; Sawning et al., 2017)
- Short presentations (to allow multiple faculty members to talk about their area of expertise)
  - (Click et al., 2019; 2020)
- Use of adaptable/flexible formats (e.g., online modules/presentations, videos)
  - (Vance et al., 2017; Vance et al., 2018; Streed et al., 2019; Altneu et al., 2020; Barrett et al., 2021; Bi et al., 2020; Braun et al., 2017; Kidd et al., 2016; Pathoulas et al., 2021; Ufomata et al., 2020; Vance et al., 2020; Ward-Gaines et al., 2021)
- Use of interactive modalities (e.g., role-play/simulation, case-based learning, clinical exposure, group discussions, debriefing, role modelling)
  - (Click et al., 2019; Greene et al., 2017; Kidd et al., 2016; Salkind et al., 2019; Sequeira et al., 2012; Streed et al., 2019; Taylor et al., 2018; Altneu et al., 2020; Barrett et al., 2021; Biro et al., 2021; McCave et al., 2019; Stumbar et al., 2021; Park and Safer, 2018; Vance et al., 2017; Vance et al., 2018; Underman et al., 2016; Berenson et al., 2020; Bi et al., 2020; Vance et al., 2021; Ward-Gaines et al., 2021)
- Theoretical/pedagogical approach compatible with/complimentary to teach transgender health (e.g., social determinants of health, intersectionality approach, locally-tailored)
  - (Rosendale and Josephson, 2017; Kidd et al., 2016; Marshall et al., 2017; Cooper et al., 2018)
- Practical, realistic content with universal clinical relevance
  - (Roth et al., 2020; Ruud et al., 2021)
- Addition of a ‘teach the teacher’ component to training
  - (Roth et al., 2021)
- Integration in already existing curriculum
  - (Roth et al., 2021; Roth et al., 2020; Ward-Gaines et al., 2021)
- Longitudinal approach (with curricular scaffolding)
  - (Altneu et al., 2020; Berenson et al., 2020; Biro et al., 2021; Stumbar et al., 2021; Levy et al., 2021)
- Learner-centered approach
  - (Altneu et al., 2020; Biro et al., 2021; Bi et al., 2020)
- Early introduction to topic
  - (Berenson et al., 2020; Biro et al., 2021; Altneu et al., 2020; Levy et al., 2021)
- Reflexivity and Feedback
  - (Bi et al., 2020; Biro et al., 2021; Stumbar et al., 2021; Vance et al., 2021)
- Hybrid design (e.g., combination of didactic and interactive components)
  - (Stumbar et al., 2021)

*Facilitation:*

- Experienced faculty (advisor) with the topic and developing trainings/courses
  - (Braun et al., 2017; Arora et al., 2019; Biro et al., 2021; Gibson et al., 2020; Stumbar et al., 2021)
- Previous experience with simulation patients
  - (Greene et al., 2017)
- Including (and availability of) transgender presenter/actor/(simulation)patient
  - (Marshall et al., 2017; Salkind et al., 2019; Underman et al., 2016; McCave et al., 2019; Arora et al., 2019; Berenson et al., 2020; Bi et al., 2020; Biro et al., 2021; Stumbar et al., 2021; Vance et al., 2021)
- Simulation patient trained by transgender community members
  - (McCave et al., 2019)
- Multi- and transdisciplinary development and facilitation
  - (Kidd et al., 2016; McCave et al., 2019; Berenson et al., 2020; Marshall et al., 2017; Click et al., 2019)
- Facilitation by LGBTQI+ students/people
  - (Braun et al., 2017; Click et al., 2019; Taylor et al., 2018; Berenson et al., 2020; Biro et al., 2021; Levy et al., 2021)
- Facilitator training and preparation
  - (Minturn et al., 2021; Barrett et al., 2021; Levy et al., 2021; Stumbar et al., 2021)
- No background knowledge or advance preparation by facilitators needed
  - (Roth et al., 2020; Ufomata et al., 2020)
- Facilitation by students/junior doctors
  - (Roth et al., 2020; Berenson et al., 2020; Bi et al., 2020; Biro et al., 2021)
- High faculty to student ratio
  - (Altneu et al., 2020; Ward-Gaines et al., 2021)

*Audience:*

- Already existing awareness/ positive attitude/ knowledge/ interest/ (personal)experience/ understanding of importance
  - (Click et al., 2019; Cooper et al., 2018; Eriksson and Safer, 2016; Marshall et al., 2017; Park and Safer, 2018; Sawning et al., 2017; Taylor et al., 2018; Underman et al., 2016; Vance et al., 2017; Berenson et al., 2020; Click et al., 2020; Gibson et al., 2020; Levy et al., 2021; Safer and Pearce, 2013)
- Interprofessional education
  - (Ruud et al., 2021; Minturn et al., 2021; Gibson et al., 2020)
- Previous experience with (LGB)T curriculum
  - (Braun et al., 2017; Marshall et al., 2017; Park and Safer, 2018; Safer and Pearce, 2013; Streed et al., 2019; Berenson et al., 2020)
- Students/residents advocating for/requesting transgender education
  - (Click et al., 2019; Kidd et al., 2016; Marshall et al., 2017; Underman et al., 2016; Biro et al., 2021; Click et al., 2020)
- No entry requirements, no background knowledge needed
  - (Altneu et al., 2020)
- Small group size
  - (Berenson et al., 2020; Stumbar et al., 2021)

*Institutional/ Contextual:*

- Available resources, time, suitable clinical training sites, simulation centers
  - (Vance et al., 2017; Vance et al., 2018; Gibson et al., 2020; Ruud et al., 2021; Vance et al., 2020; Vance et al., 2021)
- Partnerships with (LGB)T community members or organizations for development and evaluation of session material
  - (Mahabamunuge et al., 2021; Minturn et al., 2021; Ruud et al., 2021; Stumbar et al., 2021)
- Partnerships with clinicians skilled in providing gender-affirmative care
  - (Ruud et al., 2021; Vance et al., 2020)
- Sociopolitical climate positive towards the topic
  - (Eriksson and Safer, 2016; Rosendale and Josephson, 2017; Sawning et al., 2017; Safer and Pearce, 2013; Biro et al., 2021; Gibson et al., 2020)
- Incentives
  - (Gibson et al., 2020)
- Recognition of importance of training by division/ faculty
  - (Vance et al., 2020)

**References**

- Altneu, E., Grieco, C. A., Verbeck, N., Davis, J. A. & Clinchot, D. M. (2020). LGBTQ+ Health-a Novel Course for Undergraduate Students. Med Sci Educ, 30 (2), 971-976. DOI 10.1007/s40670-020-00958-5
- Arora, M., Walker, K., Luu, J., Duvivier, R. J., Dune, T., et al. (2019). Education of the medical profession to facilitate delivery of transgender health care in an Australian health district. Aust J Prim Health. DOI 10.1071/PY19102
- Barrett, D. L., Supapannachart, K. J., Caleon, R. L., Ragmanauskaite, L., McCleskey, P., et al. (2021). Interactive Session for Residents and Medical Students on Dermatologic Care for Lesbian, Gay, Bisexual, Transgender, and Queer Patients. MedEdPORTAL, 17, 11148. DOI 10.15766/mep_2374-8265.11148
- Berenson, M. G., Gavzy, S. J., Cespedes, L., Gabrani, A., Davis, M., et al. (2020). The Case of Sean Smith: A Three-Part Interactive Module on Transgender Health for Second-Year Medical Students. MedEdPORTAL, 16, 10915. DOI 10.15766/mep_2374-8265.10915
- Bi, S., Vela, M. B., Nathan, A. G., Gunter, K. E., Cook, S. C., et al. (2020). Teaching Intersectionality of Sexual Orientation, Gender Identity, and Race/Ethnicity in a Health Disparities Course. MedEdPORTAL, 16, 10970. DOI 10.15766/mep_2374-8265.10970
- Biro, L., Song, K. & Nyhof-Young, J. (2021). First year medical student experiences with a clinical skills seminar emphasizing sexual and gender minority population complexity. Can Med Educ J, 12 (2), e11-e20. DOI 10.36834/cmej.70496
- Block, L., Ha, N., Pleak, R. R. & Rosenthal, D. W. (2020). LGBTQIA+ health care: Faculty development and medical student education. Med Educ, 54 (11), 1055-1056. DOI 10.1111/medu.14312
- Braun, H. M., Garcia-Grossman, I. R., Quinones-Rivera, A. & Deutsch, M. B. (2017). Outcome and Impact Evaluation of a Transgender Health Course for Health Profession Students. LGBT Health, 4 (1), 55-61. DOI 10.1089/lgbt.2016.0119
- Click, I. A., Mann, A. K., Buda, M., Rahimi-Saber, A., Schultz, A., et al. (2019). Transgender health education for medical students. Clin Teach, 17 (2), 190-194. DOI 10.1111/tct.13074
- Click, I. A., Mann, A. K., Buda, M., Rahimi-Saber, A., Schultz, A., et al. (2020). Transgender health education for medical students. Clin Teach, 17 (2), 190-194. DOI 10.1111/tct.13074
- Cooper, M. B., Chacko, M. & Christner, J. (2018). Incorporating LGBT Health in an Undergraduate Medical Education Curriculum Through the Construct of Social Determinants of Health. MedEdPORTAL, 14, 10781. DOI 10.15766/mep_2374-8265.10781
- Eriksson, S. E. & Safer, J. D. (2016). Evidence-Based Curricular Content Improves Student Knowledge and Changes Attitudes Towards Transgender Medicine. Endocr Pract, 22 (7), 837-41. DOI 10.4158/EP151141.OR
- Gibson, A. W., Gobillot, T. A., Wang, K., Conley, E., Coard, W., et al. (2020). A Novel Curriculum for Medical Student Training in LGBTQ Healthcare: A Regional Pathway Experience. J Med Educ Curric Dev, 7, 2382120520965254. DOI 10.1177/2382120520965254
- Greene, R. E., Hanley, K., Cook, T. E., Gillespie, C. & Zabar, S. (2017). Meeting the Primary Care Needs of Transgender Patients Through Simulation. J Grad Med Educ, 9 (3), 380-381. DOI 10.4300/JGME-D-16-00770.1
- Kidd, J. D., Bockting, W., Cabaniss, D. L. & Blumenshine, P. (2016). Special-"T" Training: Extended Follow-up Results from a Residency-Wide Professionalism Workshop on Transgender Health. Acad Psychiatry, 40 (5), 802-6. DOI 10.1007/s40596-016-0570-7
- Levy, A., Prasad, S., Griffin, D. P., Ortega, M. & O'Malley, C. B. (2021). Attitudes and Knowledge of Medical Students Towards Healthcare for Lesbian, Gay, Bisexual, and Transgender Seniors: Impact of a Case-Based Discussion With Facilitators From the Community. Cureus, 13 (8), e17425. DOI 10.7759/cureus.17425
- Mahabamunuge, J., Morel, K., Budrow, J., Tounkel, I., Hart, C., et al. (2021). Increasing medical student confidence in gender and sexual health through a student-initiated lecture series. J Adv Med Educ Prof, 9 (4), 189-196. DOI 10.30476/JAMP.2021.90099.1398
- Marshall, A., Pickle, S. & Lawlis, S. (2017). Transgender Medicine Curriculum: Integration Into an Organ System-Based Preclinical Program. MedEdPORTAL, 13, 10536. DOI 10.15766/mep_2374-8265.10536
- McCave, E. L., Aptaker, D., Hartmann, K. D. & Zucconi, R. (2019). Promoting Affirmative Transgender Health Care Practice Within Hospitals: An IPE Standardized Patient Simulation for Graduate Health Care Learners. MedEdPORTAL, 15, 10861. DOI 10.15766/mep_2374-8265.10861
- Minturn, M. S., Martinez, E. I., Le, T., Nokoff, N., Fitch, L., et al. (2021). Early Intervention for LGBTQ Health: A 10-Hour Curriculum for Preclinical Health Professions Students. MedEdPORTAL, 17, 11072. DOI 10.15766/mep_2374-8265.11072
- Park, J. A. & Safer, J. D. (2018). Clinical Exposure to Transgender Medicine Improves Students' Preparedness Above Levels Seen with Didactic Teaching Alone: A Key Addition to the Boston University Model for Teaching Transgender Healthcare. Transgend Health, 3 (1), 10-16. DOI 10.1089/trgh.2017.0047
- Pathoulas, J. T., Blume, K., Penny, J., Mansh, M., Rubin, N., et al. (2021). Effectiveness of an Educational Intervention to Improve Medical Student Comfort and Familiarity With Providing Gender-Affirming Hormone Therapy. Fam Med, 53 (1), 61-64. DOI 10.22454/FamMed.2021.612374
- Rosendale, N. & Josephson, S. A. (2017). Residency Training: The need for an integrated diversity curriculum for neurology residency. Neurology, 89 (24), e284-e287. DOI 10.1212/WNL.0000000000004751
- Roth, L. T., Catallozzi, M., Soren, K., Lane, M. & Friedman, S. (2021). Bridging the Gap in Graduate Medical Education: A Longitudinal Pediatric Lesbian, Gay, Bisexual, Transgender, Queer/Questioning Health Curriculum. Acad Pediatr, 21 (8), 1449-1457. DOI 10.1016/j.acap.2021.05.027
- Roth, L. T., Friedman, S., Gordon, R. & Catallozzi, M. (2020). Rainbows and "Ready for Residency": Integrating LGBTQ Health Into Medical Education. MedEdPORTAL, 16, 11013. DOI 10.15766/mep_2374-8265.11013
- Ruud, M. N., Demma, J. M., Woll, A., Miller, J. M., Hoffman, S., et al. (2021). Health History Skills for Interprofessional Learners in Transgender and Nonbinary Populations. J Midwifery Womens Health, 66 (6), 778-786. DOI 10.1111/jmwh.13278
- Safer, J. D. & Pearce, E. N. (2013). A simple curriculum content change increased medical student comfort with transgender medicine. Endocr Pract, 19 (4), 633-7. DOI 10.4158/EP13014.OR
- Salkind, J., Gishen, F., Drage, G., Kavanagh, J. & Potts, H. W. W. (2019). LGBT+ Health Teaching within the Undergraduate Medical Curriculum. Int J Environ Res Public Health, 16 (13). DOI 10.3390/ijerph16132305
- Sawning, S., Steinbock, S., Croley, R., Combs, R., Shaw, A., et al. (2017). A first step in addressing medical education Curriculum gaps in lesbian-, gay-, bisexual-, and transgender-related content: The University of Louisville Lesbian, Gay, Bisexual, and Transgender Health Certificate Program. Educ Health (Abingdon), 30 (2), 108-114. DOI 10.4103/efh.EfH_78_16
- Sequeira, G. M., Chakraborti, C. & Panunti, B. A. (2012). Integrating Lesbian, Gay, Bisexual, and Transgender (LGBT) Content Into Undergraduate Medical School Curricula: A Qualitative Study. Ochsner J, 12 (4), 379-82. <https://www.ncbi.nlm.nih.gov/pubmed/23267268>
- Streed, C. G., Jr., Hedian, H. F., Bertram, A. & Sisson, S. D. (2019). Assessment of Internal Medicine Resident Preparedness to Care for Lesbian, Gay, Bisexual, Transgender, and Queer/Questioning Patients. J Gen Intern Med, 34 (6), 893-898. DOI 10.1007/s11606-019-04855-5
- Stumbar, S. E., Garba, N. A., Stevens, M., Gray, E., Uchimaya, E., et al. (2021). Using a Hybrid Lecture and Small-Group Standardized Patient Case to Teach the Inclusive Sexual History and Transgender Patient Care. South Med J, 114 (1), 17-22. DOI 10.14423/SMJ.0000000000001197
- Taylor, A. K., Condry, H. & Cahill, D. (2018). Implementation of teaching on LGBT health care. Clin Teach, 15 (2), 141-144. DOI 10.1111/tct.12647
- Thompson, H., Coleman, J. A., Iyengar, R. M., Phillips, S., Kent, P. M., et al. (2020). Evaluation of a gender-affirming healthcare curriculum for second-year medical students. Postgrad Med J, 96 (1139), 515-519. DOI 10.1136/postgradmedj-2019-136683
- Ufomata, E., Eckstrand, K. L., Spagnoletti, C., Veet, C., Walk, T. J., et al. (2020). Comprehensive Curriculum for Internal Medicine Residents on Primary Care of Patients Identifying as Lesbian, Gay, Bisexual, or Transgender. MedEdPORTAL, 16, 10875. DOI 10.15766/mep_2374-8265.10875
- Underman, K., Giffort, D., Hyderi, A. & Hirshfield, L. E. (2016). Transgender Health: A Standardized Patient Case for Advanced Clerkship Students. MedEdPORTAL, 12, 10518. DOI 10.15766/mep_2374-8265.10518
- Vance, S. R., Jr., Buckelew, S. M., Dentoni-Lasofsky, B., Ozer, E., Deutsch, M. B., et al. (2020). A Pediatric Transgender Medicine Curriculum for Multidisciplinary Trainees. MedEdPORTAL, 16, 10896. DOI 10.15766/mep_2374-8265.10896
- Vance, S. R., Jr., Dentoni-Lasofsky, B., Ozer, E., Deutsch, M. B., Meyers, M. J., et al. (2021). Using Standardized Patients to Augment Communication Skills and Self-Efficacy in Caring for Transgender Youth. Acad Pediatr, 21 (8), 1441-1448. DOI 10.1016/j.acap.2021.05.010
- Vance, S. R., Jr., Deutsch, M. B., Rosenthal, S. M. & Buckelew, S. M. (2017). Enhancing Pediatric Trainees' and Students' Knowledge in Providing Care to Transgender Youth. J Adolesc Health, 60 (4), 425-430. DOI 10.1016/j.jadohealth.2016.11.020
- Vance, S. R., Jr., Lasofsky, B., Ozer, E. & Buckelew, S. M. (2018). Teaching paediatric transgender care. Clin Teach, 15 (3), 214-220. DOI 10.1111/tct.12780
- Ward-Gaines, J., Buchanan, J. A., Angerhofer, C., McCormick, T., Broadfoot, K. J., et al. (2021). Teaching emergency medicine residents health equity through simulation immersion. AEM Educ Train, 5 (Suppl 1), S102-S107. DOI 10.1002/aet2.10680
